# Localization of BDNF mRNA with the Huntington's disease protein in rat brain

**DOI:** 10.1186/1750-1326-5-22

**Published:** 2010-05-27

**Authors:** Bin Ma, Brady P Culver, Gabriele Baj, Enrico Tongiorgi, Moses V Chao, Naoko Tanese

**Affiliations:** 1 Department of Microbiology, New York University School of Medicine, New York, NY 10016, USA; 2 University of Trieste, BRAIN Centre for Neuroscience, Department of Life Sciences, Via Giorgieri, 10, 34127 Trieste, Italy; 3 Molecular Neurobiology Program, Departments of Cell Biology; Physiology and Neuroscience; and Psychiatry, Kimmel Center at Skirball Institute of Biomolecular Medicine, New York University School of Medicine, New York, NY 10016, USA

## Abstract

**Background:**

Studies have implicated reduced levels of brain-derived neurotrophic factor (BDNF) in the pathogenesis of Huntington's disease. Mutant huntingtin (Htt) protein was previously reported to decrease BDNF gene transcription and axonal transport of BDNF. We recently showed that wild-type Htt is associated with the Argonaute 2 microRNA-processing enzyme involved in gene silencing. In dendrites, Htt co-localizes with components of neuronal granules and mRNAs, indicating that it might play a role in post-transcriptional processing/transport of dendritic mRNAs.

**Results:**

We conducted imaging experiments in cultured cortical neurons to demonstrate the co-localization of endogenous Htt and BDNF mRNA in fixed cells, and co-trafficking of BDNF 3'UTR mRNA with endogenous and fluorescently tagged Htt in live neurons. We used an enhanced technique that combines FISH and immunofluorescent staining to co-localize BDNF mRNA with Htt, Ago2, CPEB and dynein in thick vibratome sections of the rat cortex.

**Conclusions:**

In cultured neurons and sections of the rat cortex, we found BDNF mRNA associated with Htt and components of neuronal RNA granules, which are centers for regulating RNA transport and local translation. Htt may play a role in post-transcriptional transport/targeting of mRNA for BDNF, thus contributing to neurotrophic support and neuron survival.

## Background

Huntington's disease (HD) protein huntingtin (Htt) is a 350 kDa protein widely expressed at high levels in the hippocampus, cortex, cerebellum and striatum. Expansion of a triplet CAG repeat sequence in the 5' end of the Htt gene generates a protein with poly-glutamine repeat expansion, which is the cause of HD, an autosomal dominant neurodegenerative disorder characterized by uncontrolled movements, personality changes, dementia and death [reviewed in [1,2]]. Although the pathogenesis of HD involves many processes, current evidence suggests significant dysfunction of neurons leading to progressive neuronal loss initially in the striatum. The ubiquitous expression of Htt does not provide an explanation for the selective striatal cell neurodegeneration at the onset of HD. Wild-type Htt has been implicated in many cellular functions including regulation of gene expression, endocytosis and microtubule-directed vesicular trafficking in axons and dendrites [reviewed in [3]]. Several studies have linked brain-derived neurotrophic factor (BDNF) with HD [reviewed in [4]], and hence, it is a possible therapeutic target for the disease [5,6]. Transcription of BDNF is reported deregulated in HD, and transport of BDNF secretory vesicles necessary for neuronal survival requires a functional Htt [7,8].

Recently, we have localized Htt with the small RNA-associated protein Argonaute 2 (Ago2) in processing (P)-bodies from somatic cells [9], and in neuronal RNA granules involved in transport and local translation of mRNA in dendrites [10]. Because Ago2 has specific roles in RNA processing and gene silencing in restricted domains of the cell, the association with Htt in ribonucleoprotein particles (P-bodies and neuronal granules) provides a possible mechanism to account for the transport and translation of specific mRNAs. There are many potential genes that may be controlled at a post-transcriptional level by Ago2 and Htt. Because multiple isoforms of BDNF mRNA are transported to dendrites [11,12], we hypothesized that Htt, in association with Ago2, might regulate BDNF mRNA processing and/or trafficking.

## Results

### Huntingtin co-localizes with BDNF mRNA in cortical neurons

We recently reported that Htt associates with components of neuronal RNA granules and contributes to transport of mRNA in dendrites [10]. Purification of endogenous Htt from mouse brain extracts demonstrated presence of Ago2 protein as well as brain-specific mRNAs such as IP_3_R1, CaMKIIα and MAP2. The BDNF gene encodes for multiple alternatively spliced transcripts that target different dendritic compartments [13]. Significantly, the G196A mutation in the BDNF gene associated with neuropsychiatric disorders was found to block dendritic targeting by altering the binding site for the RNA binding protein translin which mediates dendritic targeting [13]. Because BDNF levels are reduced in the brains of HD patients and HD mouse models, we sought to investigate whether Htt might be involved in the transport of BDNF mRNA through neuronal RNA granules.

**Figure 1:**
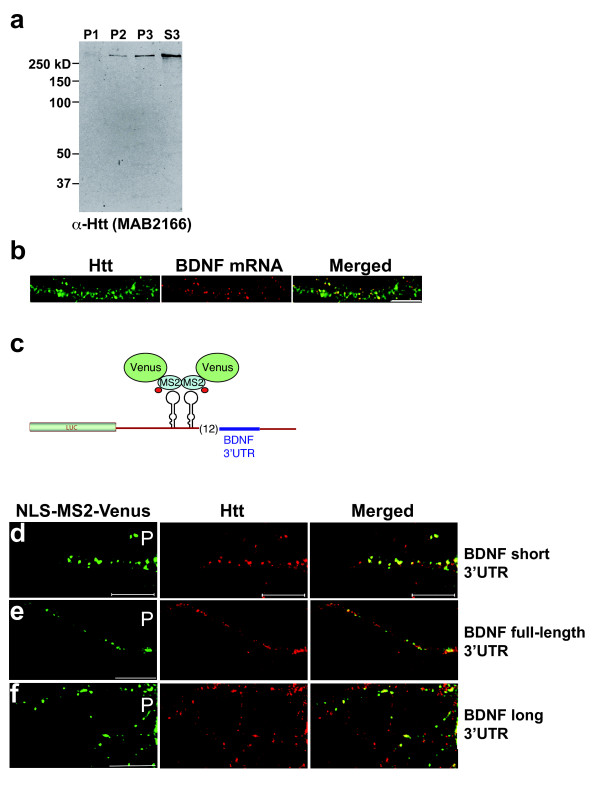
**Co-localization of endogenous Htt with BDNF mRNA in rat cortical neurons**. **(a) **Monoclonal α-Htt antibody (MAB2166, Millipore) is highly specific and demonstrates the broad subcellular distribution of full length Htt. Western blot of mouse brain fractions prepared by sequential centrifugation of total brain homogenate (see Methods) and probed with MAB2166. Htt is present in nuclear (P1), membrane-associated (P2), large cytoplasmic particles (P3), and soluble pools (S3) of protein. This broad distribution of Htt protein is in agreement to previously published observations seen with a different antibody (rabbit antibody AP78 against N-terminal peptide, [42]). **(b) **Co-localization of endogenous Htt (green) with endogenous BDNF mRNA (red) in DIV 8 rat cortical neurons. Scale bar: 10 μm. **(c) **A schematic of the MS2-based mRNA detection system. BDNF-3'UTR positioned downstream of MS2 binding sites is detected by the fluorescently tagged NLS-MS2-Venus protein by microscopy. **(d-f) **Co-localization of endogenous Htt with BDNF 3'UTR mRNA in rat cortical neurons. BDNF 3'UTR linked to MS2 is detected by the co-expressing NLS-MS2-Venus, which is shown in green, and endogenous Htt in red (Cy3). Htt co-localizing with BDNF mRNA appears yellow in merged images. BDNF mRNA reporter construct with short 3'UTR (400 bases), long 3'UTR (2.6 kb) and full-length 3'UTR (3 kb) are shown in **(d)**, **(e) **and **(f)**, respectively. Scale bar: 10 μm, P: proximal.

To localize Htt and BDNF mRNA, rat cortical neurons (DIV 8) were stained for endogenous Htt with an antibody to Htt (MAB2166, Millipore). The specificity of the antibody was first determined by western blotting of mouse brain fractions prepared by sequential centrifugation of total brain homogenate (see Methods). Probing with MAB2166 demonstrated highly specific and broad subcellular distribution of endogenous full-length Htt (Fig. 1a). Furthermore, knockdown of Htt by shRNA resulted in loss of the immunoreactive band, and reduced immunostaining of Htt in cortical neurons [10]. Htt-/- ES cells showed no detectable immunoreactivity when stained with MAB2166 [10]. Therefore, we find MAB2166 antibody to be highly specific to endogenous Htt in rodent neurons. We next detected BDNF mRNA in cortical neurons by fluorescent in situ hybridization (FISH) using the BDNF coding sequence as probe (Fig. 1b). The BDNF mRNA was visualized in the form of RNA granules, which may contain multiple (up to 30) RNA molecules [14,15]. We found BDNF mRNA to co-localize with Htt in granules, consistent with our previous finding that Htt is present in neuronal RNA granules and co-localizes with mRNAs [10].

### Co-localization of Htt with BDNF-3'UTR mRNA in cortical neurons

**Figure 2:**
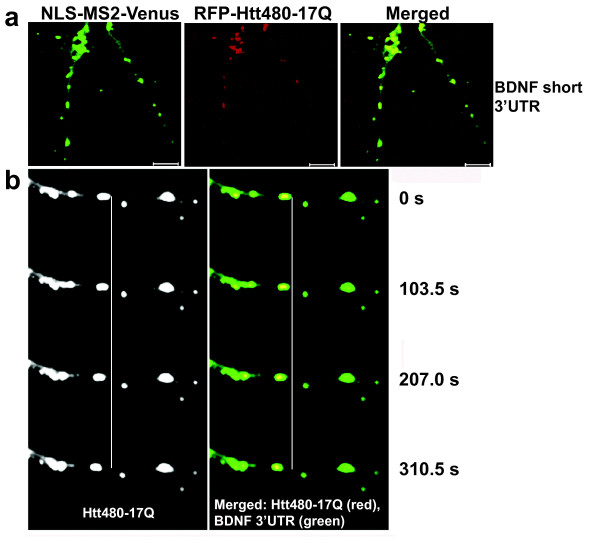
**Co-trafficking of Htt with BDNF 3'UTR mRNA in rat cortical neurons**. **(a) **The BDNF mRNA reporter plasmid with short 3'UTR was co-transfected with plasmids expressing NLS-MS2-Venus and RFP-Htt480-17Q and examined by live-cell imaging. BDNF mRNA is shown in green (detected by NLS-MS2-Venus) and Htt in red. Htt co-localizing with BDNF mRNA is in yellow in merged images. **(b) **Four cropped images from a time-lapse series in (a) captured over 310.5 seconds. Htt is shown in grayscale and the merged image in yellow. Vertical white line is drawn through the four panels to highlight the movement of one mRNA granule (left of the white line) over time. The distance that the granule traveled was 1.76 μm. Scale bar: 10 μm.

To examine further the association of Htt with BDNF mRNA in neurons, we used the MS2-fluorescent reporter RNA localization system to test if endogenous Htt can be detected in RNA granules that contain BDNF-3'UTR mRNA (Fig. 1c). This system has been used previously to investigate the role of an RNA-binding protein in dendritic transport of target mRNA [16]. Cortical neurons in culture were transfected with NLS-MS2-Venus, and an RNA localization reporter plasmid expressing BDNF-3'UTR fused to the binding sequence (MS2bs) of the bacteriophage MS2 protein. The BDNF 3'UTR region generates alternative polyadenylated mRNAs, one ending at 0.35 kb and another at 2.85 kb downstream of the stop codon [17]. A previous study reported differential localization of the two BDNF mRNA populations in neurons; however, the basis for this phenomenon remains unclear [18]. We examined three RNA reporter constructs containing full-length (2.85 kb), short (0.35 kb) and long (2.5 kb) BDNF 3'UTR sequence. Staining for endogenous Htt showed significant co-localization with all three BDNF-3'UTR reporters (detected by NLS-MS2-Venus) in cortical dendrites (Fig. 1d-f). Quantitative analysis of one projection from the image stack shows 35.7% of short 3'UTR, 38.8% of long 3'UTR and 31.5% of full-length 3'UTR to co-localize with endogenous Htt. This result suggests that Htt co-localizes with BDNF-3'UTR mRNA in dendrites. We did not detect significant differences among the reporters containing different lengths of the BDNF 3'UTR sequence. Using live-cell imaging, we also examined the localization of transfected RFP-Htt480-17Q and BDNF-3'UTR short mRNA (detected by NLS-MS2-Venus) in cortical neurons (Fig. 2). Htt was found to co-traffic with BDNF 3'UTR short mRNA in granules moving from the periphery towards the cell soma, indicating that Htt may be involved in retrograde dendritic trafficking of BDNF mRNA through its 3'UTR (Fig. 2 and additional file 1).

### Improved method to co-localize endogenous proteins with RNA in brain tissue

To study the co-localization of Htt and associated proteins with specific mRNAs in the brain, we used immunofluorescence staining (IFS) and FISH. Because of the problems in applying both FISH and IFS to thick vibratome-generated sections of rat brain tissue, and the limited sensitivity and high background of most methods, we employed a new approach using three-dimensional (3D) reconstruction on serial confocal images to visualize mRNA and proteins at high resolution. This 3D reconstruction avoids time-consuming and tedious tissue section processing and provides an accurate distribution of mRNA and proteins while retaining their status *in situ*.

A series of optimization and troubleshooting steps, described in Table 1, was carried out to maximize the signal detection in tissue sections. For combined FISH and IFS of thick vibratome sections, one challenge is to improve tissue permeabilization conditions to allow effective diffusion of probes for greater access to target mRNA and antigen. We found treatment with 0.25% Triton X-100 overnight at 4°C, or 2 hours at room temperature was effective in enhancing permeability of the sections (up to 30 μm) resulting in the immunodetection of intracellular proteins such as Ago2 and dynein. To detect low copy mRNAs (minimal 10-20 copies) in neurons, we developed a detection system utilizing polyclonal chicken α-digoxigenin (DIG) and goat α-chicken antibodies. We found that the use of tyramide signal amplification to enhance signal detection [18,19] required multiple blocking and incubation steps and displayed low resolution. In our method, FISH detection of BDNF mRNA could be performed with protein detection simultaneously without additional steps. We have utilized our improved approach to study the co-localization of the proteins Htt, Ago2, cytoplasmic polyadenylation element-binding protein 1 (CPEB1) and dynein, with BDNF mRNA as described below.

**Table 1:** Troubleshooting Guidelines

Problem	Possible reason	Solution
No or weak hybridization signal	Probe degradation	Check probe on DNA gel. If necessary, synthesize new probe.
	
	Probe concentration too low	Use more probe. Use longer hybridization time.
	
	mRNA degradation	Optimize perfusion and post-fixation conditions. Ensure that solutions, containers and instruments are RNase-free.
	
	Inadequate immunofluorescent detection of DIG-labeled probes	Titrate anti-hapten antibody. Replace with new antibody. Replace with more sensitive detection system (e.g. polyclonal anti-hapten). Use brighter fluorescent dye conjugated antibodies (e.g. Dylight).

FISH signal detected on section surface only	Poor permeabilization	Use longer incubation time or optimize detergent concentration. Use thinner sections. Use shorter probe or oligonucleotide probe.

Non-specific and/or high background staining	Probe not well purified	Optimize purification steps.
	
	Hybridization conditions not optimal	Reduce time of hybridization. Reduce probe amount. Increase time and volume of post-hybridization washes. Increase concentration of serum in blocking steps. Use 4°C for incubation with secondary antibodies. Use gentle shaking for hybridization, washing steps. Avoid sections drying out.
	
	Hybridization of probes to unwanted mRNAs	Use coding sequence only instead of entire plasmid or shorter sequence for probe generation. Use sequence-specific probes. Use oligonucleotide probes.
	
	Immunofluorescent detection of DIG-labeled probes not optimal	Adjust concentration of anti-DIG and/or anti-chicken antibodies. Spin antibody before use. Use another detection system.

### Visualization of Htt, Ago2 with BDNF mRNA

**Figure 3:**
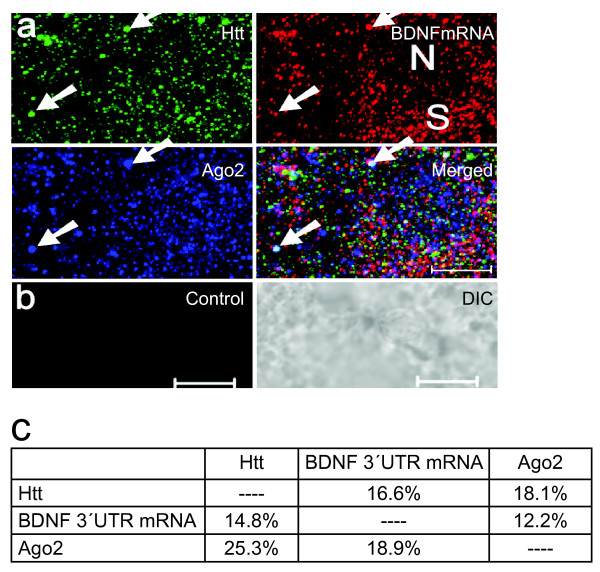
**Visualization of BDNF mRNA with Htt and Ago2 proteins in vibratome sections of a P11 rat brain cortex**. **(a) **A 3D projection of serial optical sections obtained by confocal microscopy. BDNF mRNA, Htt and Ago2 are shown in red, green and blue, respectively. Reconstruction method: LSM software, optical slice interval: 0.5 μm, stack size: 6.0 μm, N: nucleus, S: soma. White arrow indicates one co-localizing puncta of BDNF mRNA with Htt and Ago2. Scale bar: 5 μm. **(b) **Control FISH with a nick-translated probe generated from a control plasmid. DIC: differential interference contrast. Scale bar: 5 μm. **(c) **Percent co-localization of BDNF 3'UTR mRNA with Htt and Ago2. 16.6% and 18.1% of Htt was found to co-localize with BDNF mRNA and Ago2, respectively. 14.8% and 12.2% of BDNF mRNA was found to co-localize with Htt and Ago2, respectively.

Due to the localization of BDNF mRNA in somato-dendritic compartments in the hippocampus and cortex [20,21] and its prominent relationship with Htt, we utilized new enhancements to carry out both FISH and IFS together. To detect BDNF mRNA, DIG-labeled DNA probes were generated from a plasmid containing BDNF 3'UTR (see Methods). After hybridization, vibratome sections were probed with α-Htt and α-Ago2 antibodies. A representative 3D projection image is shown in Fig. 3a. No FISH signal was observed with probes generated from the control vector only plasmid (Fig. 3b). Using the co-localization analysis function of the Zeiss LSM software, we quantified percent co-localization of BDNF mRNA, Htt and Ago2 proteins in the P11 rat brain cortex (Fig. 3c). We found co-localization percentage for each pair of proteins and protein-mRNA ranging between 12 and 25%.

### Seven-color segmentation

**Figure 4:**
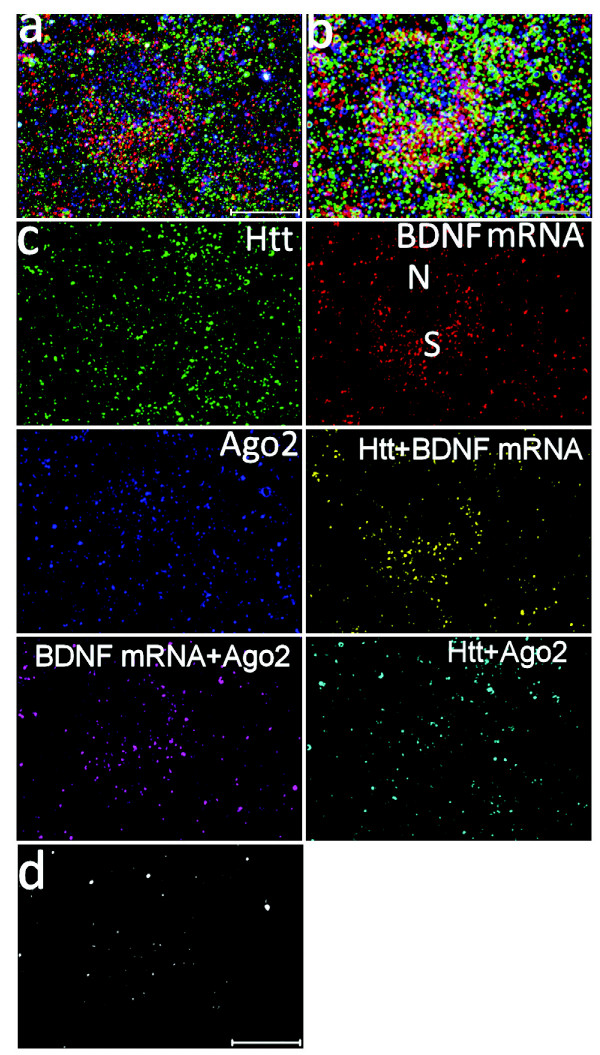
**Seven-color segmentation of a composite confocal image (3D projection) to analyze mRNA/protein and protein/protein co-localization in a rat brain slice**. Scale bar: 10 μm. **(a) **Composite image (merged from three channels) taken from Fig. 3a. BDNF mRNA, Htt and Ago2 are shown in red, green and blue, respectively. **(b) **An artificial presentation of (a) to demonstrate distribution of different color pixels in (a). **(c) **Image in (a) was segmented using self-programmed software integrated in ImageJ to generate seven pseudo channels, each of which represents single or co-localized target(s). The secondary colors yellow, cyan and magenta indicate overlap of green (Htt) and red (mRNA), green (Htt) and blue (Ago2), red (mRNA) and blue (Ago2), respectively. The seventh pseudo channel is shown in (d). N: nucleus, S: soma. **(d) **White signal is the color addition product of three primary colors green, red and blue, representing co-localization of Htt, BDNF mRNA and Ago2. Due to the limitations in the co-localization analysis function of the LSM software for confocal microscope, we can calculate percent localization for double but not triple co-localization.

Combined multicolor FISH and IFS represent a powerful way of visualizing the spatial and temporal relationship between mRNA and proteins in histological sections. However, for better visualization and interpretation of the image data, an automatic color segmentation method is needed. Because showing seven colors in one composite image is difficult for further analysis, color segmentation should be ideally made to generate six or seven pseudo channels, each of which representing a specific or a co-localizing target. In this study, a vector-based seven-color segmentation approach was applied on one composite image (Fig. 4a-b) from a 3D projection and the seven pseudo-channels were generated (Fig. 4c-d). The yellow channel represents co-localizing puncta of BDNF mRNA and Htt, the magenta channel co-localizing puncta of BDNF mRNA and Ago2, and the cyan channel co-localizing Htt and Ago2. The seventh pseudo channel is shown in panel d: white signal is the color addition product of three primary colors green, red and blue, representing the co-localization of Htt, BDNF mRNA and Ago2. Thus, this method offers visualization of the *in situ *location of protein pairs or protein and RNA from a single image.

### Visualization of Htt, CPEB1, dynein with BDNF mRNA

**Figure 5:**
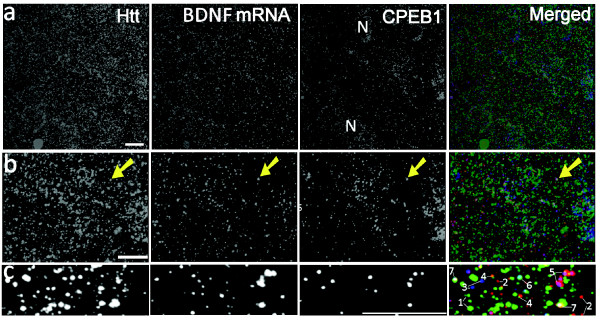
**Visualization of BDNF mRNA with Htt and mRNA-binding protein CPEB1 in vibratome sections of a rat brain cortex**. **(a) **In merged panels, BDNF mRNA, Htt and CPEB1 were imaged in red, green and blue, respectively. The three left hand columns are presented in black and white. Reconstruction method: ImageJ, optical slice interval: 0.5 μm, stack size: 16.0 μm, rotation angle: 0°. N: Nucleus **(b) **Image stack in (a) was cropped for high-resolution 3D reconstruction. Yellow arrow indicates one co-localizing puncta of BDNF mRNA with Htt and CPEB1. Co-localized spots appear white in the merged panel. N: Nucleus. **(c) **High-resolution 3D presentation of (a) to demonstrate spatial relationship of Htt/BDNF mRNA/CPEB1. Rotation angle: 170°. Htt, BDNF mRNA and CPEB1 correspond to 1, 2, 3, respectively. Co-localization of Htt/mRNA (yellow), mRNA/CPEB1 (magenta), Htt/CPEB1 (cyan) and Htt/mRNA/CPEB1 (white) correspond to 4, 5, 6, 7, respectively.

To extend these findings, the expression of BDNF mRNA was analyzed with Htt and CPEB1, a protein responsible for recognition of cytoplasmic polyadenylation elements (CPEs) and regulating poly (A) length. Representative reconstruction results are shown in Fig. 5a-b and additional file 2. The 3D temporal and spatial relationship and the co-localization of individual BDNF mRNA-containing granules with Htt and CPEB1 in vibratome sections were demonstrated by high-resolution reconstruction (Fig. 5c and additional file 3). Quantitative analysis of one projection from the CPEB1 image stack was as follows: 37.6% of BDNF mRNA co-localized with CPEB1, 17.1% of CPEB1 co-localized with BDNF mRNA, 20.0% of Htt co-localized with CPEB1, and 22.0% CPEB1 co-localized with Htt. These results confirm that Htt and BDNF mRNA are in close proximity to the machinery involved in RNA processing.

Previous investigations indicated that Htt and dynein subunits directly interact [22,23], and that Htt plays a critical role in the transport of intracellular vesicles and proteins [24]. Although transport of BDNF protein occurs in an anterograde and retrograde manner [25], there is little information with regard to the localization of BDNF mRNA in structures associated with microtubule-based molecular motors. To confirm the interaction of Htt and dynein and extend the analysis to BDNF mRNA, we carried out nick-translation of the BDNF coding sequence DNA in order to detect BDNF mRNA in rat brain sections. The sections were subsequently probed with α-Htt and α-dynein (HC) antibodies.

**Figure 6:**
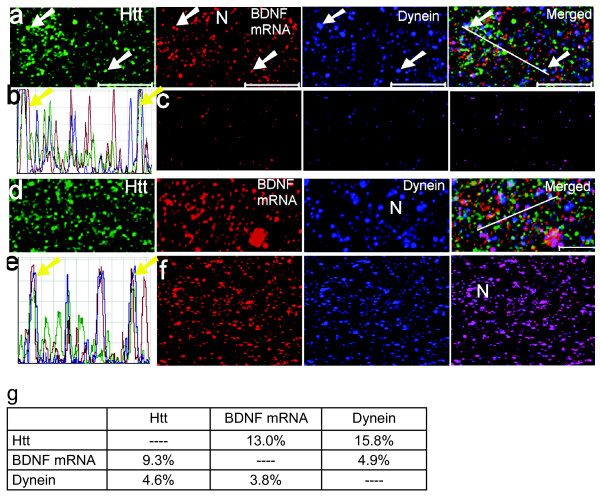
**Visualization of BDNF mRNA with Htt and dynein in vibratome sections of a rat brain cortex**. White arrow indicates co-localization of BDNF mRNA with Htt and dynein in the images, while yellow arrow indicates co-localization of BDNF mRNA with Htt and dynein in the diagram. Co-localized spots appear white in the merged panel. N: Nucleus. **(a) **An optic slice from confocal microscopy. BDNF mRNA, Htt and dynein are shown in red, green and blue, respectively. Scale bar: 10 μm. **(b) **A diagram showing intensity profile of pixels underlying the white line drawn in the merged panel in (a). **(c) **Co-localization analysis of BDNF mRNA and dynein using the LSM software. **(d) **3D projection of image stack of 37 optical slices. Reconstruction method: LSM software, optical slice interval: 0.5 μm, stack size: 18.0 μm, rotation angle: 0°. **(e) **Intensity profile of pixels underlying the white line drawn in the merged panel in (d). **(f) **3D co-localization analysis of mRNA and dynein in image stack. The pixels in red channel (mRNA) and blue channel (dynein) were segmented using a 2D thresholding approach of the whole stack and segmented stacks were reconstructed using the LSM software. One projection at rotation angle 0°is shown. **(g) **Percent co-localization of BDNF mRNA (exon 2c, 4, 6, 8, 9) with Htt and dynein.

One representative image of a two-dimensional (2D) optic slice is shown in Fig. 6a-b. The results of co-localization analysis of BDNF mRNA and dynein are shown in Fig. 6c. Using the same method described for Ago2 (Fig. 3), the co-localizing puncta of Htt/mRNA/dynein were quantified (Fig. 6g). We found 9.3% of BDNF mRNA to co-localize with Htt and 4.6% of dynein to co-localize with Htt. Using the LSM software, the image stack was reconstructed; one 3D projection is shown in Fig. 6d-e. For visualizing the co-localization events in 3D, the whole image stack was segmented and then reconstructed with the LSM software (Fig. 6f). The combinatorial approach including multicolor FISH/IFS and associated co-localization analysis indicates that Htt and dynein can be found in close proximity to BDNF mRNA.

## Discussion

Previous studies have indicated that Htt serves as a scaffold protein during the process of axonal transport of BDNF [7]. Anterograde transport of BDNF from the cortex provides trophic support to striatal neurons. Lack of cortical BDNF gives rise to atrophy and death of striatal cells [26]. In addition to axonal transport of BDNF, transcription of the BDNF gene was also reported deregulated in Huntington's disease [7,8]. Reduced BDNF levels lead to a higher susceptibility of neurons to cell death in several neurodegenerative diseases including HD [27] and Alzheimer's disease [28]. An emerging regulatory mechanism suggests that targeting of BDNF mRNA occurs in specific dendritic sites after changes in neuronal activity or high frequency stimulation [29]. Although BDNF mRNA in cortical dendrites will not reach the striatum, changes in cortical BDNF levels could affect survival of striatal cells. Dendritic translation of BDNF mRNA can regulate morphological changes in spines [12]. The rodent BDNF gene is transcribed by multiple promoters and generates at least 22 different transcripts [30,31]. The function of each mRNA isoform has yet to be characterized. One hypothesis is that different BDNF mRNAs are directed to different subcellular locations and may be locally translated and released, providing a means to selectively regulate dendritic architecture in restricted domains [21].

We previously reported that Htt co-localizes with Ago2 in processing (P)-bodies of somatic cells and neuronal granules [9,10] suggesting that Htt may play a role in RNA processing and/or gene silencing. To determine if Htt co-localizes with specific mRNAs in neuronal granules, which are structurally and functionally related to P-bodies [32], we utilized multicolor IFS and FISH techniques in concert with 3D co-localization to follow the expression of individual molecules in rat brain. We performed co-localization analysis on neurons and vibratome sections to study the interaction of proteins with BDNF mRNA. This is a number-based analysis; i.e., we analyze co-localization of BDNF mRNA granules and puncta staining of proteins. It differs from intensity-based analysis, which represents the total amount of mRNA molecules present in the granules. The discovery that BDNF mRNA is associated with Ago2 and Htt suggests a new mechanism for BDNF gene expression mediated through mRNA processing in neuronal granules.

Post-transcriptional control of BDNF mRNA in the brain likely plays a role in the production of BDNF protein [29]. While little is known about BDNF mRNA processing in neuronal granules, mechanisms of dendritic trafficking of BDNF mRNA are starting to emerge. A recent study showed that the mutation G196A (Val66Met) in the BDNF coding sequence, which is linked to impaired episodic memory and depression in humans, disrupts a recognition site in BDNF mRNA for the RNA-binding protein translin [13]. Reduced binding by translin at the 196A site was found to block trafficking of (Met)-BDNF mRNA in dendrites. Remarkably, it was previously reported that Met-carrier mice have reduced dendritic arborization and display more anxiety-like behavior similar to that of humans [33,34]. Thus, impairment of BDNF mRNA sorting and processing is likely involved in the pathogenesis of specific neuronal diseases such as HD and other psychiatric disorders. Indeed, the results shown in the present study represent a first clue that normal Htt may function in post-transcriptional repression pathways of BDNF mRNAs through P-bodies/neuronal granules and its retrograde dynein-mediated transport in dendrites. Further studies are warranted to determine how mutant Htt affects BDNF mRNA trafficking and RNA processing.

Combined multicolor FISH and IFS represent a powerful way of visualizing the spatial relationship between mRNA and proteins in histological sections. In this study, several experimental conditions have been optimized in order to perform mRNA FISH on brain tissue sections. First, brain sections need to be well fixed so that the low copy target mRNAs are protected and retained in their native location. Second, the sections need be properly processed to improve permeability, enabling probes and antibodies to reach their target. Treatments with proteinase K, sodium borohydride, ethanol gradient, or methanol have been previously applied for preprocessing tissue sections for FISH [18,35-37]; however, these steps are time-consuming and may have negative effects on signal detection. Third, to enhance the signal detection in tissue sections, methods such as tyramide signal amplification may be necessary [18,19]. Indeed, a combination of FISH and IFS may be difficult because immunodetection signal is likely weaker following FISH.

Using a number of improvements that address these shortcomings, we were able to visualize at high resolution the co-localization of BDNF mRNA with Htt and Ago2 at a single neuron level. These results suggest that Htt may play a role in post-transcriptional transport/targeting of mRNA through association with neuronal RNA granules. The targeting/trafficking of mRNA is a complex and dynamic process. The RNA-containing granules contain multiple proteins including Htt, RNA-binding proteins, motor proteins, and microtubules. Assembly/disassembly of protein/protein and protein/RNA complexes likely occur through multiple signaling pathways. Therefore, it is difficult to know whether the observed small percentage of BDNF mRNA co-localizing with Ago2 and Htt suggests involvement of a labile component. We also demonstrated the co-localization of Htt and BDNF mRNA with dynein, a motor protein involved in retrograde transport of cargos including mRNA [38]. We have also demonstrated that the 3'UTR of BDNF mRNA and Htt co-localize and co-traffic in cortical neurons. Together these findings implicate a role for Htt in maintaining neurotrophic support and neuron survival via delivery and processing of BDNF mRNA.

## Conclusions

We report the co-localization of BDNF mRNA with Htt, Ago2, CPEB and dynein in cultured cortical neurons and the rat cortex. Our combined approach of IFS/FISH and co-localization analysis provides a powerful means to study protein-mRNA interaction in neuronal cells or tissues. We show that the 3'UTR of BDNF mRNA and Htt co-localize and co-traffic in cortical neurons. These results suggest that Htt may play a role in post-transcriptional transport/targeting of mRNA through association with neuronal RNA granules. The findings implicate a role for Htt in maintaining neurotrophic support and neuron survival via delivery and processing of BDNF mRNA.

## Methods

### Brain fractionation

Brain fractions were generated as described [39]. Briefly, one flash frozen P15 Swiss Webster mouse brain was minced into a paste and homogenized on ice in a glass Dounce homogenizer in 2 ml of Buffer A (10 mM HEPES, pH 7.6; 1.5 mM MgCl_2_) containing protease inhibitors (leupeptin, pepstatin, aprotinin, PMSF, and sodium metabisulfite) and RNAse inhibitor (40 units RNAsin, Promega). The homogenate was incubated on ice for 10 minutes after which 1/10 volume of 10 × Buffer B (300 mM HEPES, pH 7.6; 1.4 M KCl, 30 mM MgCl_2_) was added. Homogenate was spun at 1,400 × *g *for 10 minutes at 4°C to pellet the P1 fraction. The P1 fractions were washed in 1 ml of 1 × Buffer B and spun as before. The combined supernatants from the 1,400 × *g *spin were spun for 20 minutes at 14,000 × *g *at 4°C to pellet the P2 fraction. The supernatant (S2) was spun for an additional 2 hours at 100,000 × *g *at 4°C to pellet the P3 fraction. Fraction P1 was resuspended in one-half the volume of total S3, fraction P2 was resuspended in same volume as total S3, and fraction P3 was resuspended in 1/10 volume of total S3. 50 mg of S3 and equivalent volumes of P1, P2, and P3 fractions were loaded onto a 7.5% SDS polyacrylamide gel for electrophoresis.

### Western blotting

50 μg of S3 and equivalent volumes of P1, P2, and P3 fractions were loaded onto a 7.5% SDS polyacrylamide gel for electrophoresis and transferred to nitrocellulose membrane, blocked for 1 hour in 5% nonfat dry milk in TBST at room temperature, and incubated in a 1:1000 dilution of MAB2166 (Millipore) in TBST overnight at 4°C. IR Dye 800 conjugated goat anti-mouse secondary antibody was used at 1:10000. Blot was scanned on a LI-COR Odyssey infrared scanner (LI-COR, Lincoln, NE).

### Preparation of nick-translated probes

DIG-labeled DNA probes were generated using DIG-Nick Translation Mix (Roche Applied Science) according to manufacturer's protocol. 1.0 μg RLTK plasmid DNA (Promega) containing BDNF 3'UTR only, or 1.0 μg of a mixture of plasmid DNA each (0.25 μg) containing exon2c, exon4, exon6, or exon8 of the BDNF gene linked to the protein coding sequence (exon9) of BDNF and fused with GFP at the 3' end [13]. The latter probe mix was used to detect "BDNF mRNA" in the current study. RLTK plasmid without any insert was used as a control. After nick-translation, Illustra ProbeQuant™ G-50 Micro Columns (GE Healthcare) were used for probe purification. 1.0 μg template DNA yielded 50 μl of probe. Probes of 200-400 bp in length were used for hybridization.

### Preparation of vibratome sections

Female wild-type Wistar rats (2 or 3 weeks old, Harlan Laboratories, Indianapolis, IN) were perfused transcardially with PBS (pH 7.4), followed by 4% paraformaldehyde (PFA, Electron Microscopy Sciences, Hatfield, PA) under deep anesthesia induced by *i.p. *injection of a mixture of ketamine (100 mg/kg) and xylazine (25 mg/kg). Brain tissues were extracted from the skull, post-fixed with 4% PFA/20% sucrose for at least two days at 4°C. All rats were maintained under veterinary supervision at New York University School of Medicine Animal Care Facility in accordance with the guidelines established by the NIH for the care of laboratory animals and all procedures approved by the Institutional Animal Care and Use Committee. 100 μm vibratome sections were prepared with a Vibratome Series 1000 Classic (Vibratome Company, St. Louis, MO) and transferred to 24-well plates filled with DEPC-PBS.

### Fluorescence in situ hybridization of mRNA

DEPC-treated water was used for preparation of PBS and other reagents. The sections were treated with 0.25% Triton X-100 in PBS overnight at 4°C and washed 3 times with PBS. After 20 minute rinsing in 1 × SSC, the sections were incubated for 2 hours at 37°C with 100 μl hybridization buffer [25% dextran sulfate, 30 μg/ml single stranded salmon sperm DNA (Sigma), 30 μg/ml yeast tRNA (Sigma), 0.4% bovine serum albumin (Jackson ImmunoResearch Laboratories), 20 mM ribonucleoside vanadyl complex (Sigma), 0.01 M sodium phosphate buffer (pH 7.0), 2 × SSC]. The sections were then hybridized for 12 hours at 37°C with 5 μl of nick-translated probe diluted in 100 μl hybridization buffer. After hybridization, sections were washed with 40% formamide/1 × SSC for 1 hour at 37°C with gentle shaking, followed by 3 × 30 minute washing with 1 × SSC with gentle shaking on an orbital shaker.

### Detection of DIG-labeled probes and immunofluorescence staining

Primary antibodies used in the study are listed in Table 2. All dilutions and washes (3 × 30 minutes) between stages were performed in PBS unless otherwise stated. Vibratome sections were washed for 20 minutes in PBS, blocked with 5% goat serum (Sigma) for 2 hours, and incubated overnight at 4°C with primary antibodies (in solution containing 5% goat serum). They were then incubated overnight at 4°C with secondary antibodies. After immunolabeling, they were transferred to Lab-Tek 2-well chamber cover glass (Nalge Nunc International) for analysis. For improved observation, a piece of cover slip (12 mm) was placed over the section to anchor it to the bottom of the chamber cover glass. Detection of DIG-labeled probes was carried out by incubating sections with chicken α-DIG antibody (Immunology Consultants Laboratory, Newberg, OR), followed by incubation with Dylight 549-conjugated goat α-chicken IgY (IgG) (Jackson ImmunoResearch Laboratories). Htt was detected by incubating sections with mouse α-Htt (Millipore), followed by Alexa 488-conjugated goat α-mouse IgG (Invitrogen). Ago2, dynein and CPEB1 were detected by incubating sections with rabbit α-Ago2 (gift of Ramin Shiekhattar, The Wistar Institute, PA), rabbit α-dynein HC (Santa Cruz Biotechnology) and rabbit α-CPEB1 (gift of David Wells, Yale University) antibodies, followed by Dylight 649-conjugated goat α-rabbit IgG (Jackson ImmunoResearch Laboratories). FISH of primary neurons was performed with a similar procedure with less processing time for incubation and washing.

**Table 2:** Antibodies used in the study

Antibody	Immunogen	Source	Host species	Isotype, Dilution used
anti-DIG	digoxigenin conjugated with KLH	Immunology Consultants Laboratory, CDIG-65A	chicken	polyclonal IgY, 1:500

anti-Htt	human huntingtin protein a.a. 181-810	Millipore, MAB2166	mouse	monoclonal, IgG1κ, clone 1HU-4C8, 1:500

anti-Ago2	human Ago2 peptide KLMRSASFNTDPYVRE	Gift of Ramin Shiekhattar, The Wistar Institute	rabbit	polyclonal, 1:1000

anti-dynein HC	rat dynein heavy chain a.a. 4320-4644	Santa Cruz Biotechnology, sc-9115	rabbit	polyclonal, IgG, 1:300

anti-CPEB1	mouse CPEB1 peptide SMEGLRHHSPLMRNQKN	Gift of David Wells, Yale University	rabbit	polyclonal, IgG, 1:1000

Alexa Fluor^® ^488 goat anti-mouse IgG	mouse IgG (H + L)	Invitrogen, A-11001	goat	polyclonal, 1:500

Cy3 goat anti-mouse IgG	mouse IgG (H + L)	Jackson ImmunoResearch, 115-166-003	goat	polyclonal, F(ab')_2 _fragment, 1:500

DyLight 549 goat anti-chicken IgY (IgG)	chicken IgY (IgG) (H + L)	Jackson ImmunoResearch, 103-505-155	goat	polyclonal, 1:500

DyLight 649 goat anti-rabbit IgG	rabbit IgG (H + L)	Jackson ImmunoResearch, 111-495-144	goat	polyclonal, 1:500

Abbreviations: DIG, digoxigenin; KLH, keyhole limpet hemocyanin; Htt, huntingtin; Ago, Argonaute; CPEB, cytoplasmic polyadenylation element binding protein

### Confocal microscopy

Confocal imaging was performed using an LSM 510 META confocal scanning laser system on an Axiovert 200 M microscope (Carl Zeiss). The instrument settings are detailed in Table 3. Images were acquired with a Plan-Apochromat 100 ×/1.3 oil-immersion objective lens, which can reduce the chromatic shift. Brightness and contrast of images were adjusted before export to Adobe Photoshop CS for further processing. Co-localization analysis was performed using the co-localization function in the LSM software. Punctate regions of fluorescence intensity are defined as focal areas of intensity greater than the average local background fluorescence plus two times the standard deviation. In Photoshop, puncta staining in neurons were counted manually in either 2D image or 3D projection image. Total events were obtained by multiplying the average number for each square by the number of squares. Percent co-localization was calculated by dividing the co-localization events by the total number of events. The following precautionary steps were taken to ensure accuracy. The results generated by the software were first compared with the original images to confirm the presence of co-localizing spots. Only a certain percentage (greater than 30%) of overlap was recognized as co-localization spots. Too little overlap of two targets, which appeared as very small spots in merged images, was not regarded as co-localization. A large sample size (more than 1500 puncta) was also used to improve accuracy of the analysis.

**Table 3:** Parameters and settings used for confocal microscopy

Fluorescent dye	Laser	Excitation wavelength (nm)	Emission filter (nm)	Detector
Alexa 488	Argon (max.12%)	488	BP 505-530	Normal

Dylight 549, Cy3	HeNe1 (max. 29%)	543	BP 560-615	Normal

Dylight 649	HeNe2 (max. 44%)	633	659-723	META

### Seven-color segmentation

Segmentation was performed using a modified version of the formerly described program [40]. The principle of this approach is in the classification of each pixel into one of seven colors (red, green, blue, yellow, magenta, cyan and white) by choosing the minimal angular deviation between the RGB vector of a given pixel and seven classically defined edge vectors. White was defined as (255, 255, 255) in RGB color model. Briefly, background was subtracted from the image and color segmentation was performed using a program integrated in ImageJ platform. After segmentation, images in seven pseudo channels were changed to grayscale and assigned seven defined colors. Finally, the images were smoothed and processed in ImageJ to generate a final version of seven pseudo channels, each of which represents one target or co-localization of two or three targets.

### 3D reconstruction

#### (a) 3D reconstruction with LSM software

The 3D projection and reconstruction were performed with Projection function in the 3D View menu. Projection method: maximum; rotation: along *y*-axis; total projections: 64.

#### (b) 3D reconstruction with Image J

ImageJ 1.42 http://rsb.info.nih.gov/ij/ was obtained from the National Institutes of Health. Stacks of images from optical sections were exported to ImageJ as serial images, processed, and saved as TIFF image sequence. 3D projections were performed using image sequence, and the movies generated were saved as uncompressed AVI files. The settings for 3D projection were: rotation angle increment: 10; opacity: 0; surface depth-cueing: 100%; interior depth-cueing: 50%; projection methods: brightest point; interpolate: selected. The slice spacing was calculated using the scale relationship of x- and z-axis. For example, if a 90 μm × 90 μm image has a size of 512 × 512 pixels and optical section interval is 0.5 μm, the slice spacing is 2.84 pixels (512 × 0.5/90).

#### (c) Compression of movie files

Movie files were compressed with Virtual Dub (Version 1.9.0.0, http://www.virtualdub.org) and saved as AVI. Cinepak Codec by Radius was used for compression.

### BDNF-3'UTR reporter plasmids

The MS2 binding sites of the bacteriophage MS2 protein were cloned downstream of the *Renilla *luciferase gene in pRL-TK vector (Promega). Full-length (2.85 kb), short (0.35 kb) or long (2.5 kb) 3'UTR sequence of the mouse BDNF gene was amplified by PCR of a genomic DNA clone (gift of Lino Tessarollo) and inserted downstream of the MS2 binding sites to generate the three BDNF-3'UTR reporter constructs.

### Transfection of primary neurons

Rat cortical neurons were isolated and cultured as previously described [41]. Neurons were fixed with 4% PFA in PBS for 20 minutes at room temperature. Rat cortical neurons of DIV5 were typically transfected with 1 μg total plasmid DNA with 1 μl of Lipofectamine 2000 (Invitrogen) in OptiMEM (Invitrogen) per 24 well as recommended by the manufacturer. Transfection of 0.5 μg NLS-MS2-Venus [16] and 0.5 μg of one of three BDNF 3'UTR constructs were used for visualization of BDNF mRNA in neurons. Before transfection, 50% of culture medium was removed and later used to replace the medium after 1-hour incubation with the transfection mixture. Neurons were fixed and immunostained 18-24 hours after transfection. Htt was detected by incubating cells with mouse α-Htt (Millipore), followed by Cy3-conjugated goat α-mouse IgG (Jackson ImmunoResearch Laboratories).

### Live cell imaging

For imaging of BDNF mRNA in live cells, 0.33 μg of NLS-MS2-Venus, 0.33 μg of a BDNF 3'UTR plasmid and 0.33 μg mRFP-Htt480-17Q [9] were transfected per well in a 24-well plate. 18-24 hours after transfection, neurons were transferred to Lab-Tek 2-well chamber cover glass (Nalge Nunc International) for live cell imaging. Less laser power (3% for Argon laser and 10% for HeNe1) was used to avoid photobleaching and toxicity. Frame time was 15 seconds and frame interval 5.0 seconds. Images were acquired with a Plan-Neofluar 40 ×/1.3 oil-immersion objective lens. The images were exported and a movie file generated by ImageJ.

## Competing interests

The authors declare that they have no competing interests.

## Authors' contributions

BM and NT conceived the study and designed the experiments, BM and BPC performed the experiments and evaluated the data, and GB and ET provided reagents. BM, ET, MVC and NT wrote the paper. All authors have read and approved the final manuscript.

## Supplementary Material

Additional file 1 **Co-trafficking of Htt with BDNF short 3'UTR in rat cortical neurons was imaged live over 610.2 seconds. BDNF 3'UTR mRNA (detected by NLS-MS2-Venus) is in green and RFP-Htt480-17Q in red.** Co-localization of Htt with BDNF 3'UTR is shown in yellow in the merged image. Click here for file

Additional file 2 **Simultaneous visualization of BDNF mRNA with Htt and mRNA-binding protein CPEB1 in vibratome sections of rat brain cortex.** BDNF mRNA, Htt and CPEB1 are shown in red, green and blue, respectively. Reconstruction method: ImageJ; optical slice interval: 0.5 μm; stack size: 16.0 μm. Click here for file

Additional file 3 **High-resolution visualization of temporal and spatial relationship and interaction of BDNF mRNA with Htt and CPEB1 in vibratome sections of rat brain cortex.** Htt, BDNF mRNA and CPEB1 are indicated with number 1, 2, 3, respectively. Co-localization of Htt/mRNA (yellow), mRNA/CPEB1 (magenta), Htt/CPEB1 (cyan) and Htt/mRNA/CPEB1 (white) are indicated with 4, 5, 6, 7, respectively. Click here for file

## References

[B1] Landles C Bates GP Huntingtin and the molecular pathogenesis of Huntington's disease. Fourth in molecular medicine review series EMBO Rep 2004 5 10 958 963 10.1038/sj.embor.7400250 15459747 PMC1299150

[B2] Li SH Li XJ Huntingtin and its role in neuronal degeneration Neuroscientist 2004 10 5 467 475 10.1177/1073858404266777 15359012

[B3] Imarisio S Carmichael J Korolchuk V Chen CW Saiki S Rose C Krishna G Davies JE Ttofi E Underwood BR Huntington's disease: from pathology and genetics to potential therapies Biochem J 2008 412 2 191 209 10.1042/BJ20071619 18466116

[B4] Zuccato C Cattaneo E Brain-derived neurotrophic factor in neurodegenerative diseases Nat Rev Neurol 2009 5 6 311 322 10.1038/nrneurol.2009.54 19498435

[B5] Gharami K Xie Y An JJ Tonegawa S Xu B Brain-derived neurotrophic factor over-expression in the forebrain ameliorates Huntington's disease phenotypes in mice J Neurochem 2008 105 2 369 379 10.1111/j.1471-4159.2007.05137.x 18086127 PMC2377033

[B6] Strand AD Baquet ZC Aragaki AK Holmans P Yang L Cleren C Beal MF Jones L Kooperberg C Olson JM Expression profiling of Huntington's disease models suggests that brain-derived neurotrophic factor depletion plays a major role in striatal degeneration J Neurosci 2007 27 43 11758 11768 10.1523/JNEUROSCI.2461-07.2007 17959817 PMC6673215

[B7] Gauthier LR Charrin BC Borrell-Pages M Dompierre JP Rangone H Cordelieres FP De Mey J MacDonald ME Lessmann V Humbert S Huntingtin controls neurotrophic support and survival of neurons by enhancing BDNF vesicular transport along microtubules Cell 2004 118 1 127 138 10.1016/j.cell.2004.06.018 15242649

[B8] Zuccato C Ciammola A Rigamonti D Leavitt BR Goffredo D Conti L MacDonald ME Friedlander RM Silani V Hayden MR Loss of huntingtin-mediated BDNF gene transcription in Huntington's disease Science 2001 293 5529 493 498 10.1126/science.1059581 11408619

[B9] Savas JN Makusky A Ottosen S Baillat D Then F Krainc D Shiekhattar R Markey SP Tanese N Huntington's disease protein contributes to RNA-mediated gene silencing through association with Argonaute and P bodies Proc Natl Acad Sci USA 2008 105 31 10820 10825 10.1073/pnas.0800658105 18669659 PMC2504805

[B10] Savas JN Ma B Deinhardt K Culver BP Restituito S Wu L Belasco JG Chao MV Tanese N A role for huntington disease protein in dendritic RNA granules J Biol Chem 2010 285 17 13142 13153 10.1074/jbc.M110.114561 20185826 PMC2857123

[B11] Tongiorgi E Righi M Cattaneo A Activity-dependent dendritic targeting of BDNF and TrkB mRNAs in hippocampal neurons J Neurosci 1997 17 24 9492 9505 9391005 10.1523/JNEUROSCI.17-24-09492.1997 PMC6573421

[B12] Tongiorgi E Baj G Functions and mechanisms of BDNF mRNA trafficking Novartis Found Symp 2008 289 136 147 discussion 147-151, 193-135full_text 18497100 10.1002/9780470751251.ch11

[B13] Chiaruttini C Vicario A Li Z Baj G Braiuca P Wu Y Lee FS Gardossi L Baraban JM Tongiorgi E Dendritic trafficking of BDNF mRNA is mediated by translin and blocked by the G196A (Val66Met) mutation Proc Natl Acad Sci USA 2009 106 38 16481 16486 10.1073/pnas.0902833106 19805324 PMC2752540

[B14] Mouland AJ Mercier J Luo M Bernier L DesGroseillers L Cohen EA The double-stranded RNA-binding protein Staufen is incorporated in human immunodeficiency virus type 1: evidence for a role in genomic RNA encapsidation J Virol 2000 74 12 5441 5451 10.1128/JVI.74.12.5441-5451.2000 10823848 PMC112028

[B15] Carson JH Cui H Barbarese E The balance of power in RNA trafficking Curr Opin Neurobiol 2001 11 5 558 563 10.1016/S0959-4388(00)00249-X 11595488

[B16] Bannai H Fukatsu K Mizutani A Natsume T Iemura S Ikegami T Inoue T Mikoshiba K An RNA-interacting protein, SYNCRIP (heterogeneous nuclear ribonuclear protein Q1/NSAP1) is a component of mRNA granule transported with inositol 1,4,5-trisphosphate receptor type 1 mRNA in neuronal dendrites J Biol Chem 2004 279 51 53427 53434 10.1074/jbc.M409732200 15475564

[B17] Timmusk T Palm K Metsis M Reintam T Paalme V Saarma M Persson H Multiple promoters direct tissue-specific expression of the rat BDNF gene Neuron 1993 10 3 475 489 10.1016/0896-6273(93)90335-O 8461137

[B18] An JJ Gharami K Liao GY Woo NH Lau AG Vanevski F Torre ER Jones KR Feng Y Lu B Distinct role of long 3' UTR BDNF mRNA in spine morphology and synaptic plasticity in hippocampal neurons Cell 2008 134 1 175 187 10.1016/j.cell.2008.05.045 18614020 PMC2527207

[B19] Pinaud R Mello CV Velho TA Wynne RD Tremere LA Detection of two mRNA species at single-cell resolution by double-fluorescence in situ hybridization Nat Protoc 2008 3 8 1370 1379 10.1038/nprot.2008.115 18714305

[B20] Pattabiraman PP Tropea D Chiaruttini C Tongiorgi E Cattaneo A Domenici L Neuronal activity regulates the developmental expression and subcellular localization of cortical BDNF mRNA isoforms in vivo Mol Cell Neurosci 2005 28 3 556 570 10.1016/j.mcn.2004.11.010 15737745

[B21] Chiaruttini C Sonego M Baj G Simonato M Tongiorgi E BDNF mRNA splice variants display activity-dependent targeting to distinct hippocampal laminae Mol Cell Neurosci 2008 37 1 11 19 10.1016/j.mcn.2007.08.011 17919921

[B22] Li SH Gutekunst CA Hersch SM Li XJ Interaction of huntingtin-associated protein with dynactin P150Glued J Neurosci 1998 18 4 1261 1269 9454836 10.1523/JNEUROSCI.18-04-01261.1998 PMC6792727

[B23] Caviston JP Ross JL Antony SM Tokito M Holzbaur EL Huntingtin facilitates dynein/dynactin-mediated vesicle transport Proc Natl Acad Sci USA 2007 104 24 10045 10050 10.1073/pnas.0610628104 17548833 PMC1891230

[B24] Colin E Zala D Liot G Rangone H Borrell-Pages M Li XJ Saudou F Humbert S Huntingtin phosphorylation acts as a molecular switch for anterograde/retrograde transport in neurons Embo J 2008 27 15 2124 2134 10.1038/emboj.2008.133 18615096 PMC2516882

[B25] Adachi N Kohara K Tsumoto T Difference in trafficking of brain-derived neurotrophic factor between axons and dendrites of cortical neurons, revealed by live-cell imaging BMC Neurosci 2005 6 42 10.1186/1471-2202-6-42 15969745 PMC1180452

[B26] Baquet ZC Gorski JA Jones KR Early striatal dendrite deficits followed by neuron loss with advanced age in the absence of anterograde cortical brain-derived neurotrophic factor J Neurosci 2004 24 17 4250 4258 10.1523/JNEUROSCI.3920-03.2004 15115821 PMC6729276

[B27] Zuccato C Cattaneo E Role of brain-derived neurotrophic factor in Huntington's disease Prog Neurobiol 2007 81 5-6 294 330 10.1016/j.pneurobio.2007.01.003 17379385

[B28] Nagahara AH Merrill DA Coppola G Tsukada S Schroeder BE Shaked GM Wang L Blesch A Kim A Conner JM Neuroprotective effects of brain-derived neurotrophic factor in rodent and primate models of Alzheimer's disease Nat Med 2009 15 3 331 337 10.1038/nm.1912 19198615 PMC2838375

[B29] Tongiorgi E Activity-dependent expression of brain-derived neurotrophic factor in dendrites: facts and open questions Neurosci Res 2008 61 4 335 346 10.1016/j.neures.2008.04.013 18550187

[B30] Liu QR Lu L Zhu XG Gong JP Shaham Y Uhl GR Rodent BDNF genes, novel promoters, novel splice variants, and regulation by cocaine Brain Res 2006 1067 1 1 12 10.1016/j.brainres.2005.10.004 16376315

[B31] Pruunsild P Kazantseva A Aid T Palm K Timmusk T Dissecting the human BDNF locus: bidirectional transcription, complex splicing, and multiple promoters Genomics 2007 90 3 397 406 10.1016/j.ygeno.2007.05.004 17629449 PMC2568880

[B32] Barbee SA Estes PS Cziko AM Hillebrand J Luedeman RA Coller JM Johnson N Howlett IC Geng C Ueda R Staufen- and FMRP-containing neuronal RNPs are structurally and functionally related to somatic P bodies Neuron 2006 52 6 997 1009 10.1016/j.neuron.2006.10.028 17178403 PMC1955741

[B33] Chen ZY Jing D Bath KG Ieraci A Khan T Siao CJ Herrera DG Toth M Yang C McEwen BS Genetic variant BDNF (Val66Met) polymorphism alters anxiety-related behavior Science 2006 314 5796 140 143 10.1126/science.1129663 17023662 PMC1880880

[B34] Soliman F Glatt CE Bath KG Levita L Jones RM Pattwell SS Jing D Tottenham N Amso D Somerville LH A genetic variant BDNF polymorphism alters extinction learning in both mouse and human Science 2010 327 5967 863 866 10.1126/science.1181886 20075215 PMC2829261

[B35] Moon IS Cho SJ Jin I Walikonis R A simple method for combined fluorescence in situ hybridization and immunocytochemistry Mol Cells 2007 24 1 76 82 17846501

[B36] Muddashetty RS Kelic S Gross C Xu M Bassell GJ Dysregulated metabotropic glutamate receptor-dependent translation of AMPA receptor and postsynaptic density-95 mRNAs at synapses in a mouse model of fragile × syndrome J Neurosci 2007 27 20 5338 5348 10.1523/JNEUROSCI.0937-07.2007 17507556 PMC6672337

[B37] Tongiorgi E Righi M Cattaneo A A non-radioactive in situ hybridization method that does not require RNAse-free conditions J Neurosci Methods 1998 85 2 129 139 10.1016/S0165-0270(98)00123-X 9874149

[B38] Goldstein LS Yang Z Microtubule-based transport systems in neurons: the roles of kinesins and dyneins Annu Rev Neurosci 2000 23 39 71 10.1146/annurev.neuro.23.1.39 10845058

[B39] Hallett PJ Collins TL Standaert DG Dunah AW Biochemical fractionation of brain tissue for studies of receptor distribution and trafficking Curr Protoc Neurosci 2008 Chapter 1 Unit 1 16 18428670 10.1002/0471142301.ns0116s42

[B40] Ma B He F Jablonska J Winkelbach S Lindenmaier W Zeng AP Dittmar KE Six-color segmentation of multicolor images in the infection studies of Listeria monocytogenes Microsc Res Tech 2007 70 2 171 178 10.1002/jemt.20401 17177276

[B41] Osten P Srivastava S Inman GJ Vilim FS Khatri L Lee LM States BA Einheber S Milner TA Hanson PI The AMPA receptor GluR2 C terminus can mediate a reversible, ATP-dependent interaction with NSF and alpha- and beta-SNAPs Neuron 1998 21 1 99 110 10.1016/S0896-6273(00)80518-8 9697855

[B42] Sharp AH Loev SJ Schilling G Li SH Li XJ Bao J Wagster MV Kotzuk JA Steiner JP Lo A Widespread expression of Huntington's disease gene (IT15) protein product Neuron 1995 14 5 1065 1074 10.1016/0896-6273(95)90345-3 7748554

